# Correction: Characterization of an accessory plasmid of *Sinorhizobium meliloti* and its two replication-modules

**DOI:** 10.1371/journal.pone.0318411

**Published:** 2025-01-24

**Authors:** The PLOS One Staff

## Notice of Republication

This article was republished on January 16^th^, 2024, to correct an error in Fig 3 and include a previously omitted funding source. The publisher apologizes for the errors. Please download this article again to view the correct version. The originally published, uncorrected article and the republished, corrected articles are provided here for reference.

## Supporting information

S1 File Originally published, uncorrected article. (PDF)

S2 File Republished, corrected article. (PDF)
