# Supplementary figures and images for: Characterization of an accessory plasmid of Sinorhizobium meliloti and its two replication-modules
Source: PLoS One. 2023 May 18;18(5):e0285505. doi: 10.1371/journal.pone.0285505 (PMC10194956; doi:10.1371/journal.pone.0285505)

A

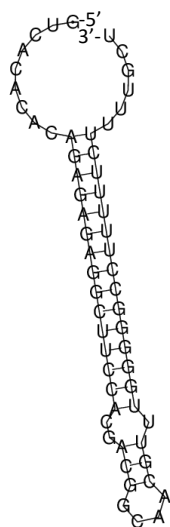

B

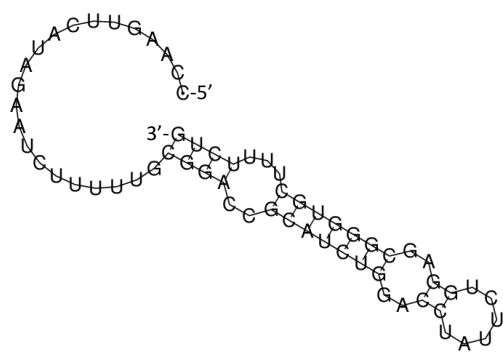

C

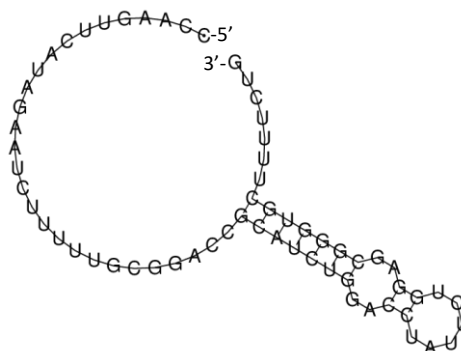

S1 Fig

Supplement: S1 Fig — A. Minimum free energy and centroid secondary structure of ctRNArepABC. B. Minimum free energy structure of ctRNArepC. C. Centroid secondary structure of ctRNArepC. (PDF) [file pone.0285505.s001.pdf]

S2 Fig

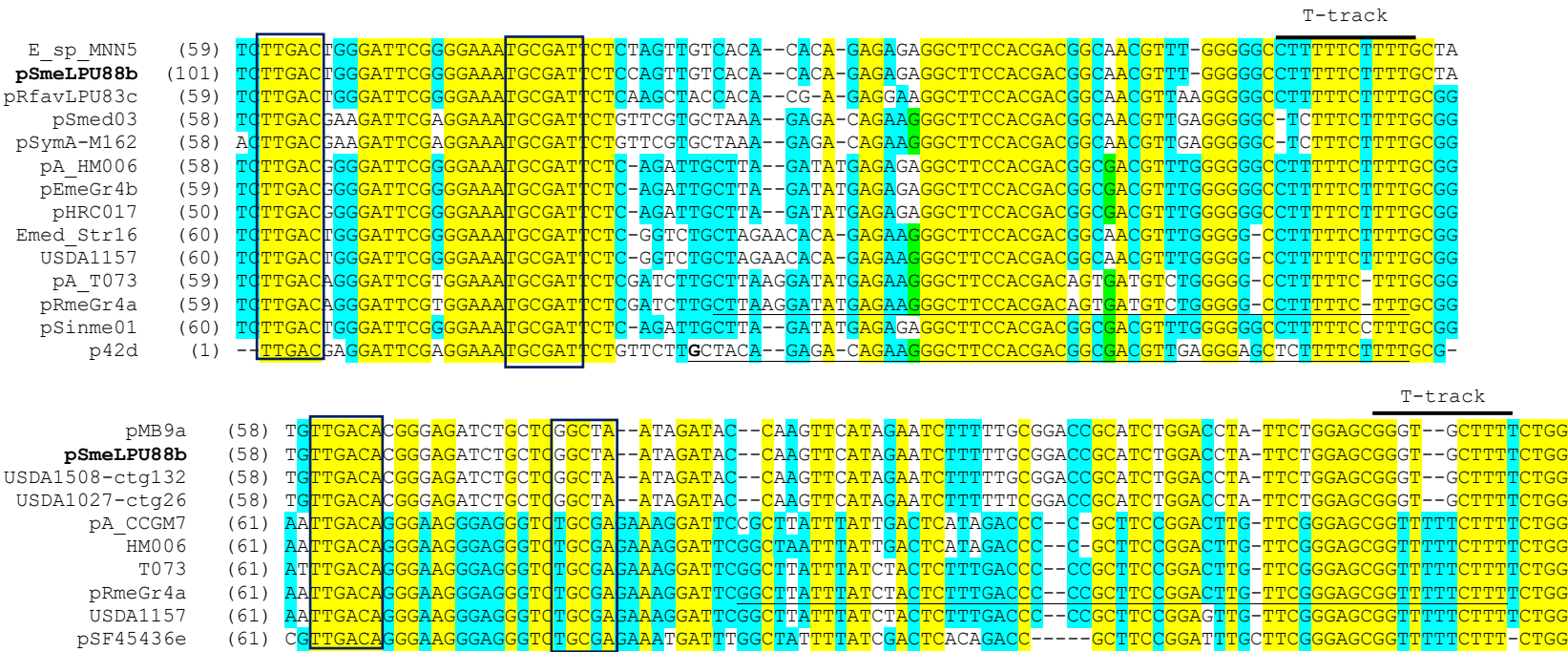

Supplement: S2 Fig — The black boxes correspond to the -10 and -35 conserved promotor region; the sequence underlined corresponds to the isolated ctRNA from plasmids pRetCFN42d and pRmeGr4a; the bold G corresponds to +1 of pRetCFN42d ctRNA. (PDF) [file pone.0285505.s002.pdf]

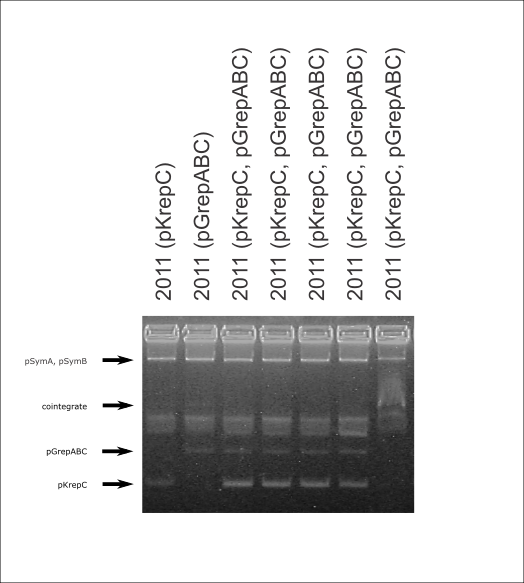

Supplement: S3 Fig — (TIFF) [file pone.0285505.s003.tiff]

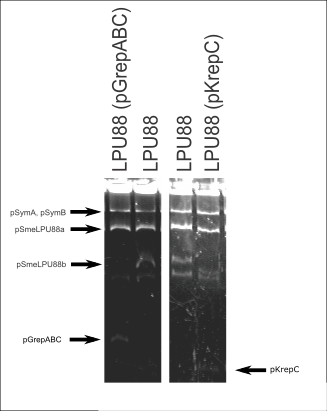

Supplement: S4 Fig — The figure shows the plasmid profile of strain LPU88 before and after receiving the constructed plasmids. (TIFF) [file pone.0285505.s004.tiff]

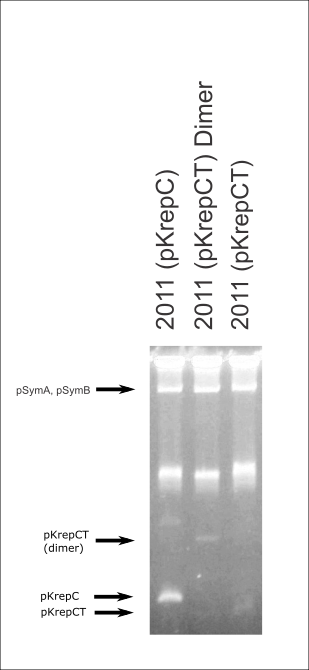

Supplement: S5 Fig — (TIFF) [file pone.0285505.s005.tiff]
