# Supplementary material for: Characterization of an accessory plasmid of Sinorhizobium meliloti and its two replication-modules
Source: PLoS One. 2023 May 18;18(5):e0285505. doi: 10.1371/journal.pone.0285505 (PMC10194956; doi:10.1371/journal.pone.0285505)
Supplement: S1 Raw images — (PDF) [file pone.0285505.s007.pdf]

# Raw images

The labels of all lanes included in the final figures are in the panel to the right of each image. Lanes that were not included in the final figure are marked with an "x".

## Fig 3A

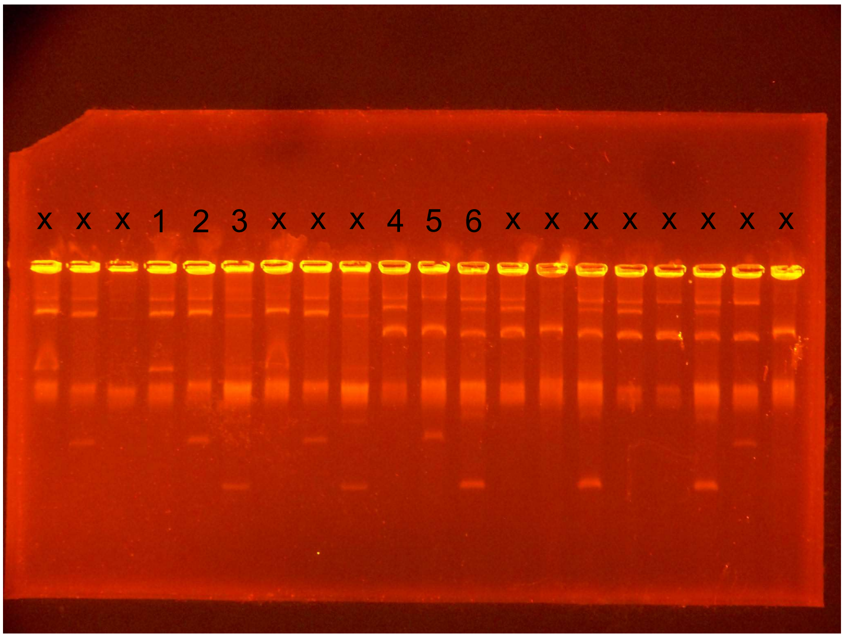


1- LPU57
2- LPU57 (pGrepABC)
3- LPU57 (pKrepC)
4- LPU178
5- LPU178 (pGrepABC)
6- LPU178 (pKrepC)

This photo was taken with Kodak Easyshare Z712 IS camera.

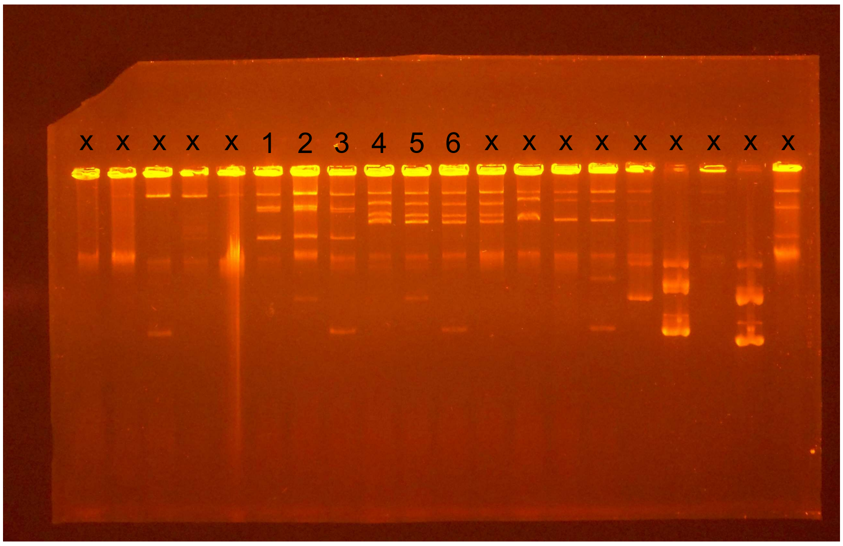


1- LPU121
2- LPU121 (pGrepABC)
3- LPU121 (pKrepC)
4- LPU122
5- LPU122 (pGrepABC)
6- LPU122 (pKrepC)

This photo was taken with Kodak Easyshare Z712 IS camera.

## Fig 3B

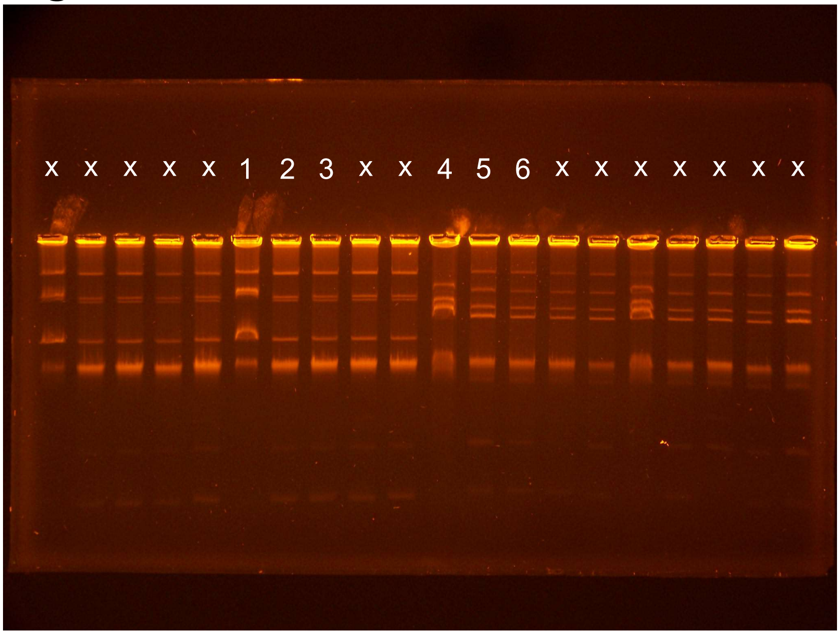


1- LPU121
2- LPU121 (pKrepC, pGrepABC)
3- LPU121 (pKrepC, pGrepABC)
4- LPU122
5- LPU122 (pKrepC, pGrepABC)
6- LPU122 (pKrepC, pGrepABC)

This photo was taken with Kodak Easyshare Z712 IS camera.

## S3 Fig

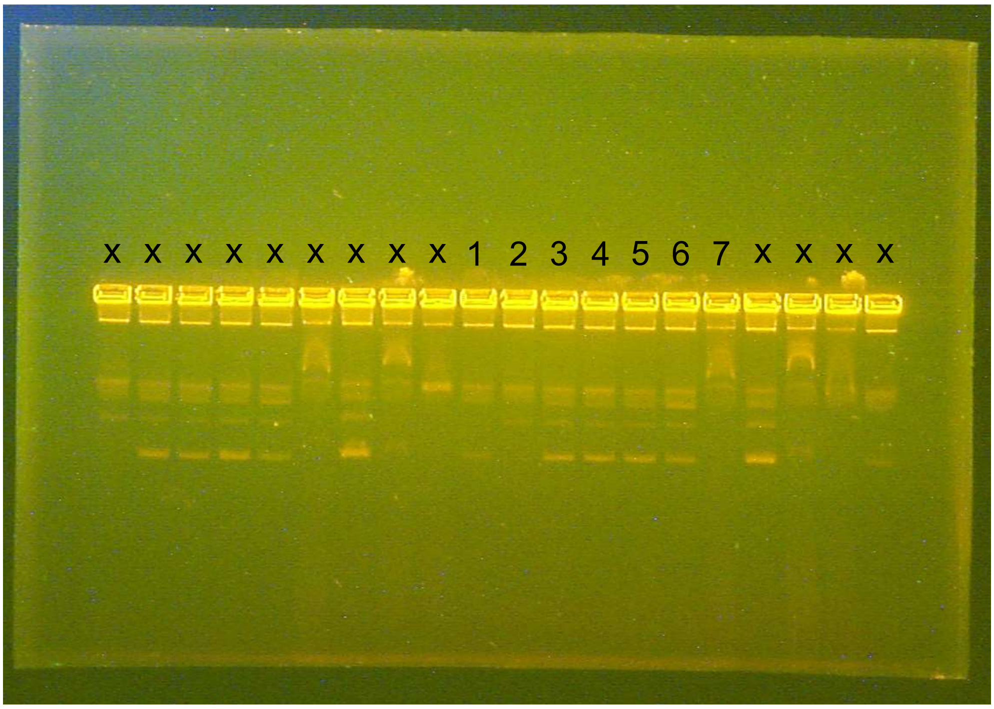


1- 2011 (pKrepC)
2- 2011 (pGrepABC)
3- 2011 (pKrepC, pGrepABC)
4- 2011 (pKrepC, pGrepABC)
5- 2011 (pKrepC, pGrepABC)
6- 2011 (pKrepC, pGrepABC)
7- 2011 (pKrepC, pGrepABC)

This photo was taken with Kodak Easyshare Z712 IS camera.

## S4 Fig

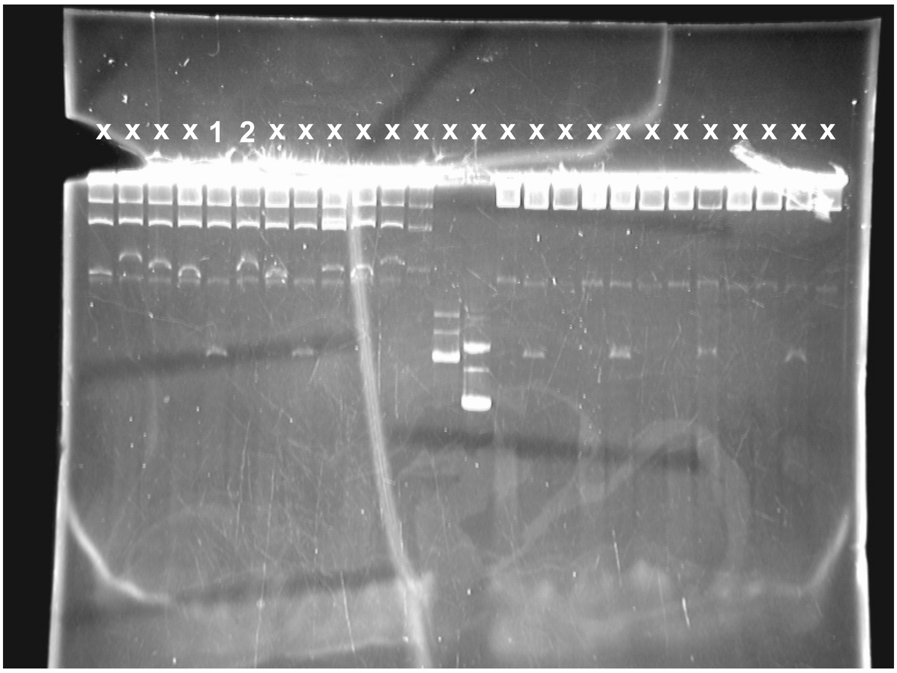


1- LPU88 (pGrepABC)
2- LPU88

This photo was taken with Pieper FK7512-IQ-IR camera.

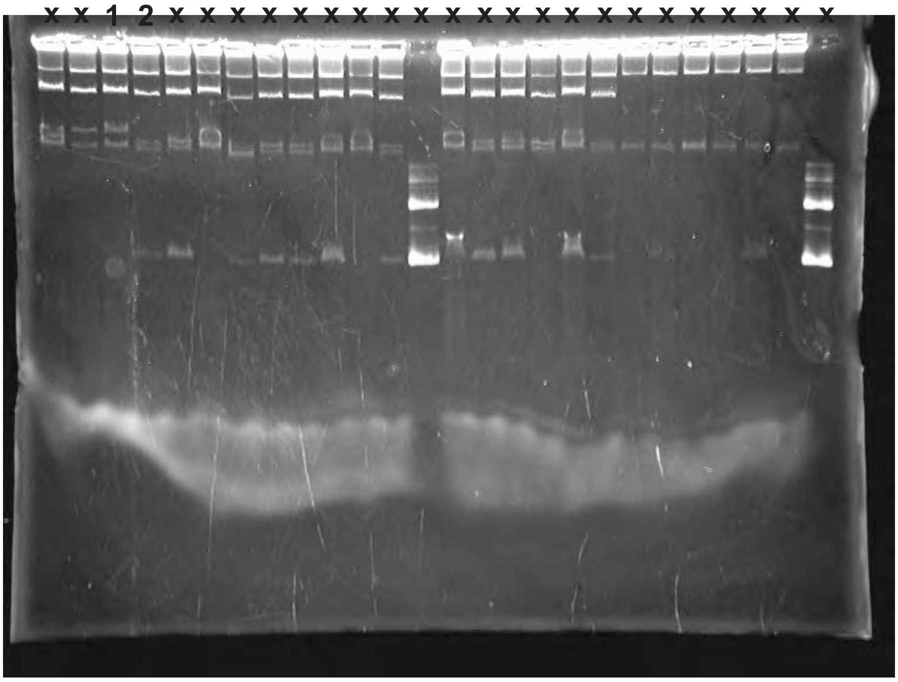


1- LPU88
2- LPU88 (pKrepC)

This photo was taken with Pieper FK7512-IQ-IR camera.

## S5 Fig

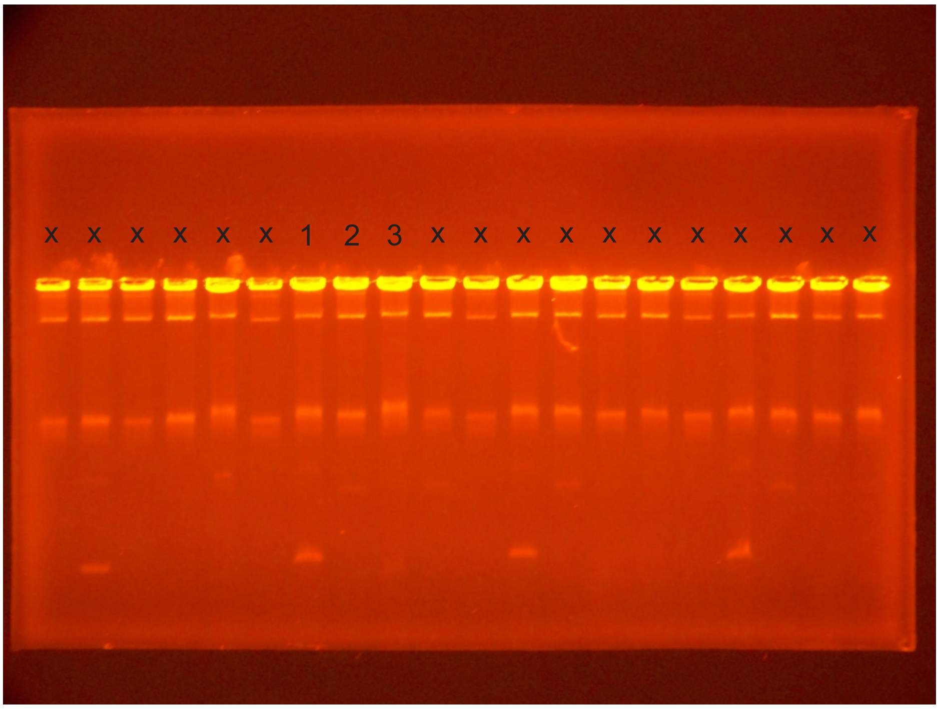


1- 2011 (pKrepC)
2- 2011 (pKrepCT) Dimer
3- 2011 (pKrepCT)

This photo was taken with Kodak Easyshare Z712 IS camera.
