# Supplementary material for: Correction: Characterization of an accessory plasmid of Sinorhizobium meliloti and its two replication-modules
Source: PLoS One. 2025 Jan 24;20(1):e0318411. doi: 10.1371/journal.pone.0318411 (PMC11760573; doi:10.1371/journal.pone.0318411)
Supplement: S2 File — (PDF) [file pone.0318411.s002.pdf]

PLOS ONE

RESEARCH ARTICLE

Characterization of an accessory plasmid of *Sinorhizobium meliloti* and its two replication-modules

Abril Luchetti[1,2], Lucas G. Castellani[2¤], Andrés Martin Toscani[2], Antonio Lagares[2], María Florencia Del Papa[2], Gonzalo Torres Tejerizo[2*], Mariano Pistorio[2*]

1 Proteome and Metabolome Research, Faculty of Biology, Center for Biotechnology, Bielefeld University, Bielefeld, Germany, **2** Departamento de Ciencias Biológicas, Facultad de Ciencias Exactas, IBBM (Instituto de Biotecnología y Biología Molecular), CCT-CONICET-La Plata, Universidad Nacional de La Plata, La Plata, Argentina

¤ Current address: Institute for Multidisciplinary Applied Biology (IMAB), Universidad Pública de Navarra (UPNA), Pamplona, España
* pistorio@biol.unlp.edu.ar (MP); gatt@biol.unlp.edu.ar (GTT)

Check for updates

OPEN ACCESS

Citation: Luchetti A, Castellani LG, Toscani AM, Lagares A, Del Papa MF, Torres Tejerizo G, et al. (2023) Characterization of an accessory plasmid of *Sinorhizobium meliloti* and its two replication-modules. PLoS ONE 18(5): e0285505. https://doi.org/10.1371/journal.pone.0285505

Editor: Günther Koraimann, University of Graz, AUSTRIA

Received: December 8, 2022

Accepted: April 25, 2023

Published: May 18, 2023

Copyright: © 2023 Luchetti et al. This is an open access article distributed under the terms of the Creative Commons Attribution License, which permits unrestricted use, distribution, and reproduction in any medium, provided the original author and source are credited.

Data Availability Statement: The minimal data set underlying the results described in our paper can be found under Genbank accession number MZ505104 (https://www.ncbi.nlm.nih.gov/nuccore/MZ505104).

Funding: G.T.T. - PICT2020-02314 - Agencia Nacional de Promoción de la Investigación, el Desarrollo Tecnológico y la Innovación - https://www.argentina.gob.ar/ciencia/ agencia M.P. - PICT2017-2833 - Agencia Nacional de Promoción de la Investigación, el Desarrollo Tecnológico y la

Abstract

Rhizobia are Gram-negative bacteria known for their ability to fix atmospheric N_2 in symbiosis with leguminous plants. Current evidence shows that rhizobia carry in most cases a variable number of plasmids, containing genes necessary for symbiosis or free-living, a common feature being the presence of several plasmid replicons within the same strain. For many years, we have been studying the mobilization properties of pSmeLPU88b from the strain *Sinorhizobium meliloti* LPU88, an isolate from Argentina. To advance in the characterization of pSmeLPU88b plasmid, the full sequence was obtained. pSmeLPU88b is 35.9 kb in size, had an average GC % of 58.6 and 31 CDS. Two replication modules were identified *in silico*: one belonging to the *repABC* type, and the other to the *repC*. The replication modules presented high DNA identity to the replication modules from plasmid pMBA9a present in an *S. meliloti* isolate from Canada. In addition, three CDS presenting identity with recombinases and with toxin-antitoxin systems were found downstream of the *repABC* system. It is noteworthy that these CDS present the same genetic structure in pSmeLPU88b and in other rhizobial plasmids. Moreover, in all cases they are found downstream of the *repABC* operon. By cloning each replication system in suicide plasmids, we demonstrated that each of them can support plasmid replication in the S. *meliloti* genetic background, but with different stability behavior. Interestingly, while incompatibility analysis of the cloned *rep* systems results in the loss of the parental module, both obtained plasmids can coexist together.

Introduction

Bacteria are ubiquitous microorganisms able to colonize and adapt to different environments in a very short-term period. Horizontal gene transfer (HGT) plays a key role in the adaptation process, being conjugative plasmid transfer one of the most efficient DNA exchange

Innovación - https://www.argentina.gob.ar/ciencia/agencia G.T.T - PIP0678 - Consejo Nacional de Investigaciones Científicas y Técnicas - https://www.conicet.gov.ar/ We acknowledge support for the publication costs by the Open Access Publication Fund of Bielefeld University and the Deutsche Forschungsgemeinschaft (DFG). The funders had no role in study design, data collection and analysis, decision to publish, or preparation of the manuscript.

Competing interests: The authors have declared that no competing interests exist.

mechanisms between bacteria [1]. Plasmids are widely distributed selfish genetic elements that are characterized by their autonomous replication. It has been shown that plasmids have many strategies to remain stable within host cell populations [2–4]. Plasmids consist of backbone genes that are involved in replication, stability and in some cases conjugative transfer, in addition to accessory genes that confer variable traits that might give an advantage to their hosts, such as antibiotic resistance, genes involved in the degradation of diverse carbon sources or in the establishment of pathogenesis or symbiosis [5].

Gram-negative bacteria belonging to the genera *Rhizobium*, *Sinorhizobium*, and *Mesorhizobium*, among others, can grow in the soil in free-living conditions and in symbiosis with the root of leguminous plants as nitrogen-fixing organisms. A common feature of the genomes of these genera is that, in addition to the chromosome, they usually harbor plasmids encoding widely diverse functions [6–8]. Rhizobial plasmids vary greatly in number (1 to 10) and size (ten to thousands of kilobases) and can constitute a high proportion of the bacterial genome [6, 7, 9]. Many of them have relevant roles during the interaction between rhizobia and the host plant. Most of the genes required for the symbiotic process are encoded in the so-called symbiotic plasmids or pSym [10]. In addition to these pSym, most of the plasmids carried by rhizobia are dispensable for symbiosis or have simply not yet been assigned a specific function. These plasmids with no apparent function are referred, in a generic manner, as non-symbiotic, cryptic, or accessory plasmids [7].

For the survival of plasmids in the host cell, they must replicate through a functional replication system, independent of the chromosomal replication system. In addition, different mechanisms involved in plasmid stability have been described in the literature, such as multimer resolution, active partitioning and post-segregational killing, also known as toxin-antitoxin or plasmid addiction systems [2–4]. Regarding replication systems, necessary to achieve independent replication, the best-studied system in soil bacteria is the *repABC* replication system [11]. In the Rhizobiaceae family, specifically, *repABC*-type replicons predominate in plasmids in which their replication regions have been studied [12, 13]. The *repABC* system consists of four genes: an operon composed of three genes (*repA*, *repB*, and *repC*) [14–18] and a gene encoding a small regulatory antisense RNA (ctRNA) located in the complementary strand of the intergenic space between *repB* and *repC* [19–22]. RepA and RepB belong to the ParAB family of partition proteins. The interaction between RepA, RepB and a centromere-like sequence (*parS*) provides the plasmid's segregation machinery [11, 13, 23]. The *parS* site of *repABC* plasmids consists of single or multiple 16 bp palindromic sequence that could be located at different positions in each plasmid [24–26]. Regarding RepC, it has been proposed to be the initiator protein [15]. Cervantes-Rivera et al. [27] showed that in the *repABC* operon of pRetCFN42d of *Rhizobium etli* CFN42, *repC* is the only required element to sustain replication. In addition, the origin of replication of the *repABC* plasmids is located at the central section of the *repC* gene [27, 28]. Incompatibility determinants are also commonly present in plasmids. For *repABC* plasmids, three main incompatibility determinants were described [20, 24, 25, 29–31]. In addition to being involved in plasmid segregation, the 16 bp palindromic sequence *parS* exerts strong incompatibility towards the parental plasmid or plasmids having identical *parS* [24, 25]. The other elements involved in plasmid segregation, RepA and RepB, could induce plasmid incompatibility when expressed *in trans*, downregulating the transcription of the *repABC* operon [29, 31]. Another incompatibility determinant consists of the ctRNA located between *repB* and *repC*. It has been demonstrated that the expression of this small RNA *in trans* causes the loss of the parental plasmid due to the interference with the expression of *repC* [20, 25]. Recently, Rivera-Urbalejo et al. [30] mapped the sequences of the ctRNA that are required for plasmid incompatibility by site directed mutagenesis. Besides the *repABC* system, other replication systems have been described in rhizobia. The second system

of replicons, called the *repC* family, is evolutionarily related to the *repABC* family. These systems share the replication initiation protein RepC, but in contrast to the organization of *repABC*, *repC* is not forming an operon with *repA* and *repB* genes [32]. In this family, an antisense RNA also plays a central role as a negative regulator of *repC* expression and as a determinant of incompatibility [33]. A third system, includes only one member, the 7.2 kb pRm1132f plasmid isolated from *S. meliloti* strain 1132, which also belongs to group II of rolling circle replication systems [34].

As we mentioned before, the segregational stability of the rhizobial plasmids is achieved by the activity of plasmid-specific partitioning proteins (RepAB) that direct plasmid copies to new daughter cells during cell division. In addition to partitioning systems, plasmids can harbor toxin-antitoxin (TA) genes to ensure the inheritance of plasmids. TA systems are small genetic modules coding for a stable toxin and for an unstable antitoxin protein that counteracts the activity of the toxic protein. Wheatley et al [35], described the presence of a TA module on *Rhizobium leguminosarum* bv. *viciae* 3841 pRL10 plasmid and associate its presence to the inability to lose pRL10.

In our laboratory, we have been studying rhizobial accessory plasmids as vehicles of adaptation and evolution [36–41]. We have previously described the transmissibility properties of two cryptic plasmids from the strain *Sinorhizobium meliloti* LPU88, a local isolate from Argentina. One of them, pSmeLPU88b, resulted to be mobilizable only if helper functions were supplied *in trans* by the accompanying plasmid pSmeLPU88a (binary conjugal system) [41]. Later, it was shown that the mobilization region of plasmid pSmeLPU88b presented a new mob region that was conserved in other rhizobia and in different Gram-negative bacteria [42]. To get a deeper insight into the traits encoded by this plasmid we report here the complete sequence and the genomic characterization of pSmeLPU88b. Moreover, we studied the replication and stability elements found in it. Plasmid pSmeLPU88b presents two origins of replication, one belonging to the *repABC* family and another related to the *repC* family. Interestingly, both are capable of supporting the replication of the plasmid. It is noteworthy that the pSmeLPU88b plasmid backbone is not only conserved in *S. meliloti* plasmids but also in *Sinorhizobium* spp. and *Rhizobium* spp. strains thus, our work depicts replication genes distributed in rhizobia and contributes to the basic knowledge of rhizobial plasmid biology.

Materials and methods

Bacterial strains and plasmids

The strains and plasmids used are listed in Table 1. *Escherichia coli* was grown on LB [43] medium at 37˚C. *S. meliloti* strains were grown on TY [44] medium at 28˚C. For the agarized media 15 g of agar per liter of medium was added. The growth kinetics (28˚C, 200 rpm) was analyzed by monitoring OD_{600} in a microplate reader (BMG Labtech, Germany). The final antibiotic concentrations per mL of medium were: 25 μg kanamycin (Km) and 10 μg gentamycin (Gm) for *E. coli*; 400 μg streptomycin (Sm), 120 μg neomycin (Nm), and 30 μg Gm for *S. meliloti*.

DNA sequencing and bioinformatics tools

The pSmeLPU88b plasmid sequence was obtained by sequencing *S. meliloti* LPU88 at SNPsaurus (https://www.snpsaurus.com/illumina-bacterial-assembly/) by means of Illumina technology. The reads obtained were assembled using Spades Assembler software (Version 3.12.0). Plasmid finishing was accomplished using a previously sequenced plasmid library [42]. The accession number for plasmid pSmeLPU88b is MZ505104. Plasmid pSmeLPU88b was automatically annotated by the genomes annotations platforms Prokka [48] and RAST

Table 1. Bacterial strains and plasmids used in this work.

Strain and plasmids	Relevant Properties	Source or Reference
Escherichia coli		
E. coli DH5α	*recA*, Δ*lacU169*, F80d*lac* ZDM15	Bathesda Res. Lab.
E. coli S 17–1	*E. coli* 294 RP4-2-Tc::Mu-Km::Tn7 integrated into the chromosome	[45]
Sinorhizobium meliloti		
2011	Sm^r, Nod^+ Fix^+ in alfalfa, derived from strain SU47	J. Denarié, Toulouse, France
LPU88	Wild type isolate from Argentina, Sm^r	[41]
LPU57	Wild type isolate from Argentina, Sm^r	[36]
LPU121	Wild type isolate from Argentina, Sm^r	[36]
LPU122	Wild type isolate from Argentina, Sm^r	[36]
LPU178	Wild type isolate from Argentina, Sm^r	[36]
Plasmids		
pK18mob	Km^r, high copy number cloning vector.	[46]
pBBR1MCS-2	Km^r, broad host range	[47]
pG700	Gm^r, pG18mob2,	[42]
pKmutC	pK18mob, containing an internal fragment of *repC2*	This work
pKmutABC	pK18mob, containing an internal fragment of *repC1*	This work
pKrepC	pK18mob, containing *repC* family replicon	This work
pGrepABC	pG700, containing the *repABC* family replicon	This work
pKrepC-T	pK18mob, containing a truncated *repC* family replicon	This work
pBBRparS	pBBR1MCS-2 containing a 0,7 kb fragment of pSmeLPU88b	This work

Nm^r, Sm^r, and Gm^r = neomycin, streptomycin, and gentamicin resistance, respectively.

https://doi.org/10.1371/journal.pone.0285505.t001

[49] and then manually curated. Sequence alignment and graphic comparison were performed using the genome comparison visualizer Easyfig [50]. For the identification of ctRNAs present in pSmeLPU88b plasmids, the intergenic region between the *repB* and *repC* genes of *repABC* family and the region upstream of *repC* of *repC* family were aligned along with the ctRNA genes from some *repABC* and *repC* families. The transcriptional start sites of p42d [20] and pRmeGR4a [33] have been previously experimentally determined. We used that information to map the ctRNAs. The ctRNA structures were predicted using the RNAfold WebServer (http://rna.tbi.univie.ac.at//cgi-bin/RNAWebSuite/RNAfold.cgi).

Bacterial mating

Bacterial matings were performed as described by Simon et al [45]. Stated in brief, liquid cultures were grown to the early exponential phase for donor cells and the late exponential phase for recipient cells. Donor and recipient strains were mixed in a 1:1 volume ratio and plated on TY plates, and then incubated overnight at 28˚C. Afterwards, the transconjugants and controls were plated in TY medium supplemented with the appropriated antibiotics.

Plasmid profiles—Eckhardt gels

Cells were grown in TY medium to the mid-exponential phase and the plasmid profiles were performed according to previously established techniques [51, 52].

DNA manipulation and genetic constructs

Plasmid and total DNA preparation, restriction-enzyme analysis, cloning procedures, and *E. coli* transformation were performed as previously described [53].

Construction of *repC* mutants

An internal fragment of each *repC* gene was amplified with the appropriate primers (S1 Table) with Pfu polymerase (for *repC1*: repC1-(ABC)-MN, repC1-(ABC)-MC; for *repC2*: repC2-MN repC2-MC) (S1 Table). The obtained fragment was cloned in the *SmaI* site of pK18mob (a suicide vector in rhizobia) resulting in the vectors pKrepCmut and pKrepABCmut. The obtained plasmids were transferred by conjugation to strain LPU88 to yield the site-specific insertional mutagenesis. The insertion was evaluated by PCR with external primers: repC1-(ABC)-Ext and repC2-trunc for *repC1* and *repC2*, respectively (S1 Table).

Functional analysis of replication modules

RepC family replicon. The complete coding sequence of *repC* gene and 500 bp upstream was obtained using primers repC-F and repC-R (S1 Table) amplifying a 2,492 bp fragment with Pfu polymerase. This fragment was cloned in the *SmaI* site of pK18mob (this vector is not able to replicate in rhizobia). The constructed plasmid, designed pKrepC, was introduced into *S. meliloti* strains by conjugation, using *E. coli* S17-1 as the donor, to determine if this plasmid was able to replicate in rhizobia and if it is incompatible with different plasmids.

RepABC family replicon. We have previously described the obtention of a partial library of pSmeLPU88b plasmid [42, 54]. One clone of the library, pG700, contained a DNA fragment encoding the C-terminal domain of the RepC of the *repABC* operon in pG18mob2 plasmid (non-replicative in rhizobia). pG700 was introduced into strain LPU88 by conjugation. The presence of sequences in common between pG700 and pSmeLPU88b enabled the formation of cointegrate plasmids. The entire *repABC* operon was then obtained by isolation of total DNA, digestion of isolated genomic DNA with restriction endonuclease *Sma*I, to generate a restriction fragment containing the integrated plasmid and *repABC* operon. Circularization of this DNA fragment by ligation and transformation into *E. coli* DH5α competent cells allow us to obtained plasmid pGrepABC.

Truncated RepC2. By introducing a stop codon in the reverse primer (repC2-trunc, S1 Table), a truncated variant of the *repC2* gene was amplified by PCR and cloned in the *SmaI* site of the pK18mob suicide plasmid. The position of the reverse primer was selected after aligning the DNA sequence of *repC2* of LPU88 to other *repC* family sequences.

parS site. In the non-replicative pG700 plasmid the parS site was identified. To test its function, pG700 plasmid was digested with *Eco*RI and the liberated fragment cloned in the replicative plasmid pBBR1MCS-2, yielding pBBRparS.

Stability of plasmids

The stability of plasmids in *S. meliloti* LPU88 and *S. meliloti* 2011 was determined as previously described [55]. The proportion of bacteria that harbor plasmids was determined by plating subculture steps on TY agar and further replication on TY agar plates and TY agar plates containing the corresponding antibiotics for each plasmid. Colonies that had lost the plasmid were able to grow on TY agar plates but not in TY agar plates with antibiotics, while colonies harboring plasmids were able to grow on both plates. The rate of plasmid loss was calculated.

Results and discussion

Overall features of pSmeLPU88b

We have previously reported the conjugal properties of two cryptic plasmids from the strain *S. meliloti* LPU88 [41]. To further advance in the characterization of the mobilizable plasmid pSmeLPU88b, the complete nucleotide sequence was obtained and analyzed, focusing on gene content and backbone gene organization. General features of pSmeLPU88b sequence revealed the following: the plasmid genome is 35,933 bp long and its GC content is 58.6%. The annotation of the plasmid nucleotide sequence was first done automatically by Rast [49] and Prokka [48] followed by manual curation, resulting in 31 CDS (Fig 1A, Table 2). The 31 CDS presented identity to genes in the NCBI nr database and 25 of them (78%) could be assigned to a Cluster of Orthologous Group (COG) (Table 2). Two of the remaining six CDS could be assigned to a PFAM family while four CDS could not be assigned to any specific function.

Of the total CDS, eleven were associated with plasmid biology functions, like replication (*repABC1* and *repC2*), stability (CDS 4,5,6), and mobilization (*parA*-like, *mobCZ*, *traI*).

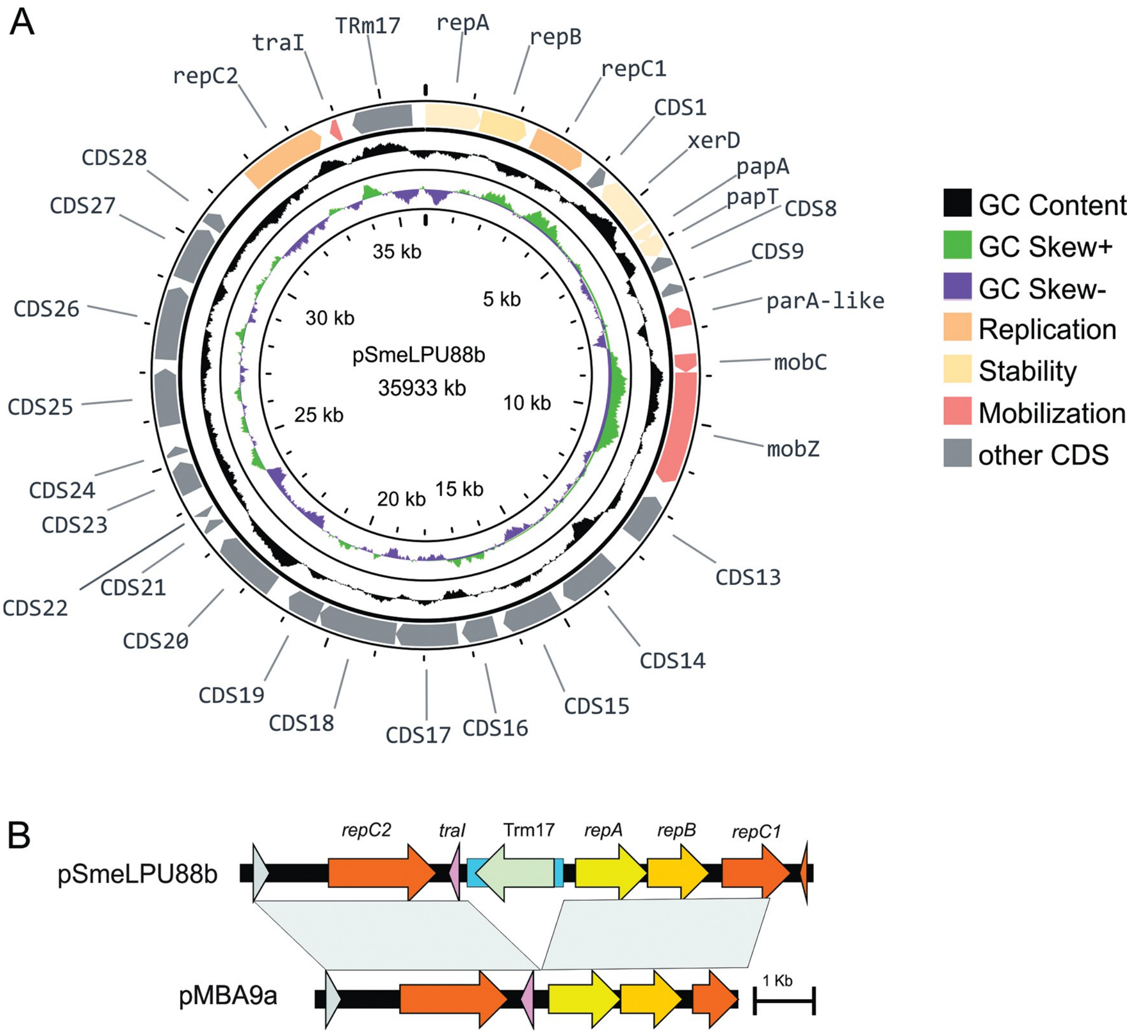

Fig 1. Genome plot and comparison of pSmeLPU88b. A. Schematic plot of pSmeLPU88b. From the inner to the outer circle: genomic position in kb; GC skew; GC content; predicted protein-coding sequences (CDS). The *repA* gene was chosen as the first gene. The plot was made using https://proksee.ca/. B. Structural comparisons of pSmeLPU88b and pMBA9a replication regions. The arrows indicate genes and the blue rectangle IS*Rm17*.

https://doi.org/10.1371/journal.pone.0285505.g001

Table 2. Protein-coding sequences identified in plasmid pSmeLPU88b.

Feature_id	Predicted Function	COG#	Protein Best Match	Identity
repA	Plasmid partition protein RepA	D	AAX19273	100%
repB	Plasmid partition protein RepB	K	AAX19274	100%
repC1	Plasmid replication protein RepC	K	WP_127529104.1	100%
CDS4	hypothetical protein		WP_127529192.1	99%
CDS5—*xerD*	Site-specific recombinase XerD	L	ASQ15018	94%
CDS6—*papA*	Phd antitoxin, type II toxin-antitoxin system Phd/YefM family antitoxin	D	ASQ15019.1	100%
CDS7—*papT*	plasmid maintenance toxin (PemK-like)		WP_127529191.1	100%
CDS8	hypothetical protein		WP_127514965.1	100%
CDS9	hypothetical protein	S	WP_127529194.1	100%
parA-like	ATPases involved in chromosome partitioning	D	AFC88005.1	92%
mobC	mobilization protein		WP_127529222.1	100%
mobZ	putative relaxase	U	WP_127652933.1	87%
CDS13	Uncharacterized conserved protein, NAD-dependent epimerase/dehydratase family	S	WP_127529155.1	100%
CDS14	Aminotransferase class I/II-fold pyridoxal phosphate-dependent enzyme	H	WP_127529156.1	100%
CDS15	Glutamate dehydrogenase/leucine dehydrogenase	E	WP_127529157.1	97%
CDS16	Transcriptional regulator, GntR family	K	WP_127529161.1	100%
CDS17	Glutamate-1-semialdehyde aminotransferase (EC 5.4.3.8)	H	WP_127529162.1	100%
CDS18	putative acetolactate synthase large subunit	EH	WP_127529158.1	99%
CDS19	3-oxoacyl-[acyl-carrier protein] reductase (EC 1.1.1.100)	IQ	WP_127529159.1	99%
CDS20	Succinate-semialdehyde dehydrogenase [NAD(P)+] (EC 1.2.1.16)	C	WP_127652730.1	97%
CDS21	hypothetical protein		WP_153499494.1	86%
CDS22	hypothetical protein	S	WP_127529111.1	100%
CDS23	Response regulator transcription factor. Phosphate regulon transcriptional regulatory protein PhoB (SphR)	KT	WP_127529110.1	
CDS24	hypothetical protein		WP_127529109.1	100%
CDS25	ATP-grasp domain-containing protein	M	WP_127529108.1	100%
CDS26	MFS permease	EGP	WP_127529107.1	100%
CDS27	ATP-grasp domain-containing protein	F	WP_127529106.1	100%
CDS28	Mobile element protein. IS110 family transposase	L	WP_127570297.1	99%
repC2	Plasmid replication protein RepC	K	WP_127529105.1	99%
traI	N-acyl-L-homoserine lactone synthetase TraI	QT	AAX19272	
TRm17	ISNCY family transposase ISRm17	L	WP_010970069.1	

COG Categories: C, Energy production and conversion; D, Cell cycle control and mitosis; E, Amino Acid metabolis and transport; F, Nucleotide metabolism and transport; G, Carbohydrate metabolism and transport; H, Coenzyme metabolism; I, Lipid metabolism; K, Transcription; L, Replication and repair; M, Cell wall/ membrane/envelop biogenesis; P, Inorganic ion transport and metabolism; Q, Secondary Structure; T, Signal Transduction; S, Function Unknown.

https://doi.org/10.1371/journal.pone.0285505.t002

Previously, Giusti et al, [42] characterized the CDS *parA*-like and *mobCZ*, and these were found to be associated with the DNA-transfer-and-replication region (Dtr) of pSmeLPU88b (Table 2). The other identified CDS encode proteins associated with different metabolic processes (CDS18, CDS19, CDS25, CDS26, CDS27) like amino acid metabolism (CDS14, CDS17) or oxidoreduction (CDS 13, CDS15, CDS19, CDS20), transcriptional regulation (CDS16, CDS23) and transposition (CDS28, TRm17) (Table 2). Five CDS encoded for hypothetical proteins (CDS4, CDS8, CDS9, CDS21, CDS22) that are also found in other *S. meliloti* strains, but for which no other inferences could be made (Table 2).

In silico analysis of the replication and stability modules

The *in silico* analysis of the obtained sequence data allowed us to identify two plasmid replication modules: one belonging to the *repABC* family [13], and the other related to *repC* family

[32]. The DNA alignment of both regions resulted in nearly identical elements to the ones present in the plasmid pMBA9a from an *S. meliloti* isolate from Canada (99% DNA identity) [56]. However, while the *repC* and *repABC* modules present in pMBA9a are contiguous, in the plasmid pSmeLPU88b are separated by the IS-element IS*Rm17*, which belongs to the group IS*Doll* of the ISCNY family (Fig 1B). The presence of more than one type of replication system in the *S. meliloti* cryptic plasmids seems to be a common feature since it has also been observed in other plasmids like pRmeGR4a, pRmeGr4b, pSmeSM11a, pHRC017, and the paccessoryA of strains HM006, T073 and USDA 1157. This feature is not only limited to *S. meliloti* species since pSF45436e from *Sinorhizobium fredii* CCBAU45436, plasmid A from *Sinorhizobium americanum* CCGM7, pRtrCIAT899b from *Rhizobium tropici* CIAT899 and pAtS4a from *Agrobacterium vitis* S4 present two types of replicons. In addition, the presence of two replicons seems to be a common feature in alphaproteobacteria. Bartosik et al. [18] described a plasmid of *Paracoccus versutus UW1* containing *repC* and *repABC* systems. Later, the same authors found several strains of *Paracoccus pantotrophus* containing four plasmids with both types of replication machinery [16].

Usually, RepC proteins from repC family (PRK13824 domain) present a size of ca. 400 amino acids long (https://www.ncbi.nlm.nih.gov/Structure/sparcle/archview.html?archid=11486891), but it is noteworthy that RepC2 from pSmeLPU88b has 611 amino acids. There are only three entrances in the NCBI database with a RepC protein with the same characteristics, all of them of *S. meliloti* strains. Specifically, in pMB9a of strain MB9A, and in DNA contigs from strains USDA 1027 and USDA 1508, all of them sharing 99% amino acid identity.

A general characteristic of *repABC* operons is the presence of a conserved intergenic region between the *repB* and *repC* genes. In this region, a gene coding a 55–59 nt small non-translated RNA (ctRNA) in the opposite orientation to the *repABC* operon is usually located, which could generate incompatibility with its parental plasmid if it is introduced *in trans* [19, 20]. A similar antisense RNA was described for the *repC* family [33]. These ctRNA not only play a role as an incompatibility factor but also as a negative regulator of *repC* gene expression [33]. We identified *in silico* ctRNA, upstream of each *repC* gene. The computer-predicted secondary structure of both ctRNA presented similar structures as those previously described (S1 Fig) [19, 20, 22, 33]. An alignment of ctRNAs of other rhizobial plasmids showed that $ctRNA_{repABC}$ presents a higher sequence conservation than $ctRNA_{repC,}$ and the last one is identical to the ctRNA present in pMB9a and the contigs from USDA 1027 and USDA 1508 strains (S2 Fig).

The centromere-like DNA sequence *parS* consist of one or more copies of a 16-bp palindromic consensus sequence `GGTNNGNGCNCNNACC` close to the *repABC* genes [13]. The *parS* locus could be located downstream from *repC* [15], within the *repA-repB* intergenic zone [57], or upstream from *repA* [25]. Recently, Czarnecki et al. [26] showed that in the *repABC* system of plasmid pAMI4 of *Paracoccus aminophilus*, in addition of a single *parS* near the *repABC* genes, the plasmid contains three additional *parS* repeats, 11.5 kb downstream of *repC*. We searched for the presence of *parS* in pSmeLPU88b and found a 16-bp palindrome, 93 bp downstream of *repC1* gene. The palindrome sequence `CCTGTCAGCTGACAGG` is partially conserved compared to the consensus sequence.

Downstream of the *repABC* replication system are located three CDS homologous to recombinases and toxin-antitoxin (TA) systems. The recombinase (CDS 5, XerD) presents its maximum amino acid identity (96%) with the protein CDO22_34430 (ASQ15018) of plasmid accessory A of *S. meliloti* HM006. The CDS 5 belongs to the Cre-like recombinases, a tyrosine-based site-specific recombinase. These enzymes are associated with plasmid maintenance by mediating the recombination site between the repeated sequences of the concatemer. Plasmid dimers could be formed through homologous recombination and, since a dimer has two origins of replication, the copy number control system perceives two entities, but only one

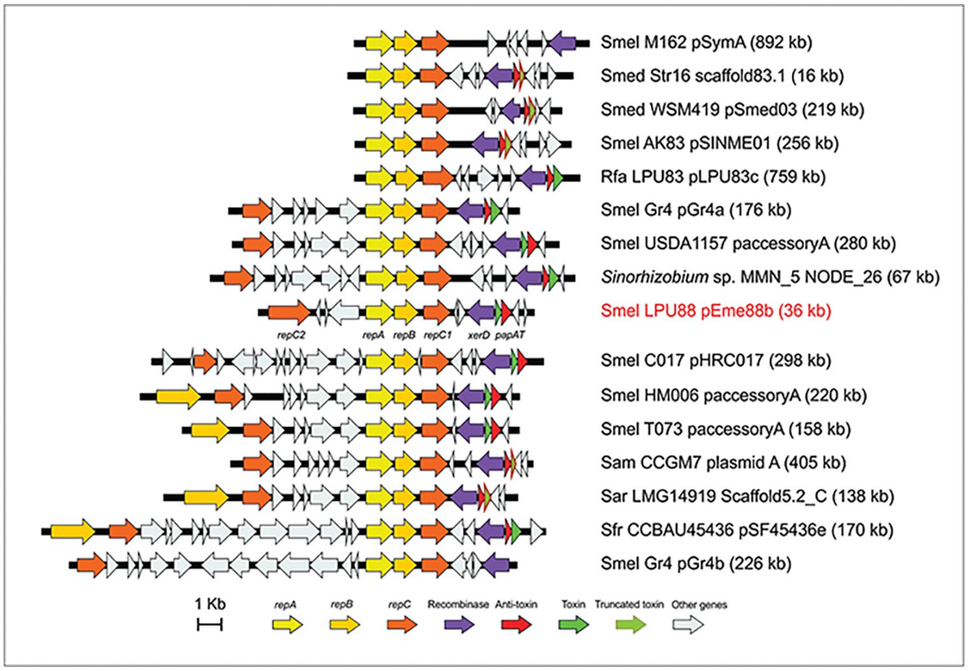

Fig 2. Comparison of replication regions. Genetic organization of pSmeLPU88b replication region compared with regions present in other rhizobial plasmids. Relevant orthologs are marked with the same color.

https://doi.org/10.1371/journal.pone.0285505.g002

plasmid is available for segregation [58]. In *Rhizobium etli* CFN42 it was described a recombinase RinQ, which is required to exert incompatibility to pRetCFN42d plasmid. RinQ is also near to the *repABC* operon but belongs to the invertase/resolvase family (γδ family) [59]. In pLPU88b, the TA system is formed by CDS 6 and CDS 7 (designated *papA* and *papT* respectively for *plasmid addiction protein*). PapA shown 100% amino acid identity with the CDO22_34435 (accessory A plasmid of *S. meliloti* HM006) and CN144_34000 (contig 185 of strain USDA 1508). PapT presented 100% amino acid identity to CN144_33995 (contig 185 of strain USDA 1508). PapA belongs to the type II toxin-antitoxin systems (pfam02604, Antitoxin Phd_YefM), members of this family bound to their toxin partners, and they can bind DNA repressing the expression of TA operons. PapT possesses a conserved putative domain belonging to the PemK superfamily, which inhibits the growth of host cells. PemK is responsible for mediating cell death by inhibiting protein synthesis through the cleavage of single-stranded RNA. This system is completed by the antitoxin PemI that inhibits the action of the PemK toxin [60]. PemI belongs to the MazE_antitoxin family (pfam04014), a family different from the PapA antitoxin family.

It is noteworthy that the *repABC* and the three CDS observed downstream presented the same genetic structure in pSmeLPU88b and in other rhizobial plasmids. Apparently, this genetic organization is a common feature in *S. meliloti*, *S. medicae*, and there are representatives in *S. fredii*, *S. americanum*, *S. arboris* and *Rhizobium favelukesii*. In addition, in some plasmids, there is also present a *repC* family replicon (Fig 2). This region could be the so-called "backbone" of a plasmid, which generally encodes functions involved in replication, maintenance, and in conjugative plasmid transfer. As we mentioned before, plasmids containing *repC* and *repABC* systems could be found in other alphaproteobacteria. The search for complete plasmid sequences in databases showed that plasmids pAK2 of *Paracoccus* sp. AK26, pBM151 of *P. tegillarcae* BM15, pZO2 of *P. zhejiangensis* J6, plasmid a of *Rhodobacter*

sphaeroides MBTLJ-20 and plasmid D of *R. sphaeroides 2.4.1*, among others, present both types of replication systems. However, in these plasmids the recombinase and TA systems are not found in the neighborhood of the *repABC* system. Furthermore, the two origins of replication were located further apart from each other compared to the distance in rhizobial plasmids.

Functional characterization of the two-replication modules of plasmid pSmeLPU88b

As we mentioned before, rhizobial strains contain several plasmids and each one of them carries genes of the *repABC* or *repC* families. The most obvious case of this peculiarity is observed in *R. etli* CFN42 and *R. leguminosarum* 3841, each of which has six plasmids all belonging to the *repABC* family. This suggests that different *repABC* plasmids belong to different incompatibility groups [61, 62]. Furthermore, replicons harboring two *repABC* operons have also been found [61, 62]. In other examples, plasmids with one *repC* and one *repABC* were observed [56, 63, 64]. This condition has been proposed as beneficial [65], assuming that the presence of more than one replication module can contribute to the overall stability of the plasmid that carries them.

To determine the functionality of the *repABC* and *repC* replicons present in pSmeLPU88b, we carried out two strategies: on the one hand, we made site-specific insertional mutagenesis on each of the replication initiation proteins; in a second approach, each of the replication modules was cloned separately in vectors that originally are suicide in *S. meliloti*, but with the replication module became replicative. In both assays, the ability to maintain replication in rhizobia was evaluated. We could obtain mutants in both *repC* genes by insertional mutagenesis, demonstrating the functionality of both replicons. After cloning each replication module in the suicide vectors, the resulting pKrepC and pGrepABC (Table 1) plasmids were transferred to *S. meliloti* 2011 and *S. meliloti* LPU88 strains. To verify the presence of plasmids pKrepC and pGrepABC in rhizobia, transconjugants were taken randomly and analyzed by Eckhardt-type gels. The replication functionality of the new build replicons has been first evaluated through the presence of the expected plasmid in *S. meliloti* 2011 (S3 Fig) and in *S. meliloti* LPU88 (S4 Fig).

Another interesting aspect to mention is the incompatibility of the vectors pKrepC and pGrepABC with respect to the plasmid pSmeLPU88b. Incompatibility between plasmids is the inability of two plasmids to coexist within the same cell. In general, this situation is the result of sharing certain elements involved in plasmid replication or segregation, as well as in some regulatory components of these functions. Strikingly, when plasmids pKrepC and pGrepABC were transferred independently by conjugation to LPU88 strain, pSmeLPU88b was removed from the cells (S4 Fig). The analysis by Eckhardt-type gels confirmed that both replicons could exclude plasmid pSmeLPU88b (S4 Fig). It should be noted that selection pressure with antibiotics -Nm (for pKrepC) and Gm (for pGrepABC)-, forces the maintenance of the introduced plasmids instead of the resident plasmid pSmeLPU88b. This result was surprising because it was expected that pSmeLPU88b would replicate by the different replication system as the one introduced. The observed behavior shows the existence of certain interactions between both replication systems. To corroborate this hypothesis, we transferred the plasmid pGrepABC to the *S. meliloti* 2011 derivative previously containing pKrepC, and the plasmid pKrepC to the *S. meliloti* 2011 derivative previously containing pGrepABC (S3 Fig). In the *S. meliloti* 2011 background, the interaction/interference between the replicons was not observed since they could replicate independently in most of the cases, but in a few cases a cointegration between pKrepC and pGrepABC was observed (S3 Fig).

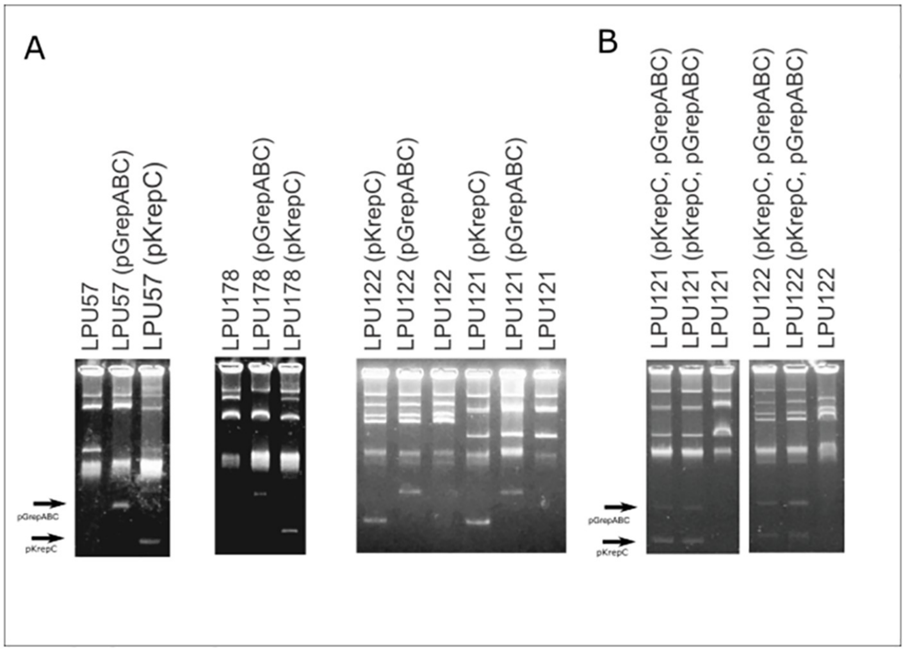

Fig 3. Plasmid profiles of isolates exhibiting incompatibility with the incoming plasmid pKrepC or pGrepABC. A. Profile of plasmids from isolates LPU57, LPU178, LPU121 and LPU122 before and after having received the replication modules of pSmeLPU88b. B. Plasmid profiles of isolates LPU122 and LPU121 harboring both pSmeLPU88b replication modules (plasmids pkrepC and pGrepABC).

https://doi.org/10.1371/journal.pone.0285505.g003

As we saw that each replication module was functional, the maintenance of the plasmids was tested. First, the *repC* mutants were evaluated. *S. meliloti* LPU88 cells carrying pSmeLPU88::pKmutC or pSmeLPU88::pKmutABC (mutants in each module) were grown in serial cultures in a nonselective medium, and plasmid loss rates were measured by plating to determine the proportion of cells retaining the plasmid (see Materials and Methods). Both plasmids were stably maintained in progeny cells since 100% of the cells retained the plasmid after four subculture steps (96 h). However, plasmid loss could be prevented due to the presence of the TA system in pSmeLPU88b. Thus, the TA could mask the effect of the *repC* mutations on plasmid replication initiation and/or plasmid segregational stability. To test this hypothesis, we evaluated the bacterial growth kinetics. The assay showed that generation time was lower in the wild type (1.83±0.06 h) compared to the mutants (2.38±0.05 h for *repABC* mutant and 2.23±0.06 h for *repC* mutant. P<0,0001 Tukey's test). The loss of pSmeLPU88b would induce the elimination of cells by the activity of TA system. These events of cell death would justify the lower growth rates observed for the mutants. These results suggest that the insertional mutations destabilize the plasmid replication, and that the TA system could be active, eliminating the cells without plasmid. Next, the stability of the two cloned replicons was separately examined in *S. meliloti* 2011. Plasmid pG18repABC showed similar stability to that of pSmeLPU88b (100%). In contrast, pKrepC was lost after 4 subcultures (96 h), where only 0.4% of cells retained the plasmid. Similar observations were described by Bartosik et al. [18] with the replicons of pTAVl plasmid.

To test the function of the identified *parS*, the plasmid pBBRparS was conjugated to LPU88 (pGrepABC) to determine if this sequence showed any incompatibility with the pGrepABC present in the strain. The analysis of the transconjugants showed that all clones only carried

the pBBRparS and not the pGrepABC plasmid, confirming the incompatibility between these two elements. Therefore, this result corroborates that the identified sequence, although divergent with the consensus, is a functional *parS* site.

Incompatibility of each replication module to other *S. meliloti* plasmids

In previous work, we analyzed the incompatibility of pSmeLPU88b within a collection of rhizobia isolates, to gain insight into how this phenomenon affects the intraspecific mobilization of *S. meliloti* plasmids [36]. The analysis showed that four isolates, LPU57, LPU178, LPU121, and LPU122, have lost at least one plasmid in presence of pSmeLPU88b, proving that this plasmid is incompatible with some plasmids of the mentioned isolates. To investigate if some of the replication modules of pSmeLPU88b present incompatibility, plasmids pKrepC and pGrepABC were transferred to each isolate and the transconjugants were analyzed by Eckhardt gels electrophoresis. The results showed that for isolates LPU57 and LPU178 only the pGrepABC plasmid exerted incompatibility (Fig 3A). However, neither pKrepC nor pGrepABC plasmids were able to eliminate the resident plasmids in the strains LPU121 and LPU122 (Fig 3A). Perhaps, the incompatibility only manifests when both replicons are present, thus, we generated strains with plasmids pKrepC and pGrepABC. As it is shown in Fig 3B, the incorporation of both plasmids to isolates LPU121 and LPU122 did not produce any change in resident plasmids. We concluded that the incompatibility of pSmeLPU88b to the resident plasmids of strains LPU121 and LPU122 is not because of the replication modules. Thus, three options arise: both replication modules should be in the same plasmid to exert incompatibility, the incompatibility could be due to segregation issues or other genes present in pSmeLPU88b may be involved. In general, plasmid incompatibility occurs when multiple plasmids within one cell have the same replication and/or partitioning system. In the case of rhizobia, as it was mentioned before, pRetCFN42d contains, in addition to replication and partitioning genes, a gene that influences plasmid stability and incompatibility properties that is not encoded by the *repABC* operon [59]. In plasmids pTi-SAKURA and pTiC58 of *Agrobacterium tumefaciens*, two TA systems were shown to enhance plasmid stability and incompatibility [66, 67]. This could be the case of pSmeLPU88b since it carries a TA module that might compensate the loss of TAs present in the plasmids of LPU121 and LPU122 strains, but pKrepC and pGrepABC were not able to do it.

Analysis of the replication initiator protein belonging to the *repC* family

Strikingly, as we mentioned before, RepC2 of plasmid pSmeLPU88b has 611 amino acids when almost all the known replication initiator proteins, RepC and RepABC families are *ca.* 400 amino acids. A protein alignment of RepC2 with other RepC proteins showed that the first 400 amino acids are conserved and present the typical Rep proteins' motif. Furthermore, a BlastX analysis showed an IS4 family transposase (only c-term 44% protein identity) coded in the complementary strand of *repC* gene 3'-end. These results support the idea that the additional 200 C-terminal amino acids of RepC2 could be the consequence of an ancient event of an IS4 element transposition.

In order to assess if only the first 400 amino acids are enough for replication, a truncated variant of the RepC was generated by PCR, introducing a stop codon in the reverse primer and cloned in the pK18mob vector. The obtained plasmid, pKrepCT, was transferred to the strain *S. meliloti* 2011 to test functionality. The presence of pKrepCT was confirmed in the plasmid profiles electrophoresis (S5 Fig), confirming that the first 400 amino acids of RepC2 are able to perform replication.

Concluding remarks

Rhizobia usually carry plasmid DNA that can reach 45% of the total amount of genetic information in that cell [9]. In many cases, the information that is harbored in these replicons contributes to the saprophytic survival or to the development of symbiosis with leguminous plants. In this work, we presented the sequence of pSmeLPU88b, an accessory mobilizable plasmid of *S. meliloti* LPU88 [41]. The sequence analysis showed a genomic structure with backbone modules for replication/partitioning and mobilization. In addition, a region carrying information for metabolic processes that could be useful for adaptation to the environment was identified.

Plasmids must replicate within the cells. The most common replication system among rhizobial plasmids is the one based on the *repABC* family [13], but also *repC* family of replicons containing only the *repC* has been described in other rhizobia [33, 68]. The presence of more than one replication module is not rare in rhizobial plasmids. Interestingly, the *repC* family is always found in plasmids carrying a *repABC* family cluster. Remarkably, pSmeLPU88b showed two functional replication modules, one of them belonging to *repABC* and the other to *repC* families. In particular, *repC2* of pSmeLPU88b (which belongs to the *repC* family) showed an atypical size, being 30% larger than the average RepC proteins. It was identified that an insertion of a mobile genetic element creates a frameshift that increase the protein size. Nevertheless, we corroborated that the first 400 amino acids are enough for the functionality of RepC2.

Despite both replicons being able to replicate, it seems that they have a different relevance. While the *repABC* remains stable, *repC2* was lost in the same period. The low stability shown by the *repC2* type of replication module in rhizobia could explain the reason why *repC* modules are always found in plasmids harboring a *repABC* module in rhizobia. In this way, the overall stability of the plasmid would be greater. Furthermore, we could speculate that *repC* modules are expressed in particular conditions to guarantee the prevalence of the plasmids. Moreover, the presence of two replicons could be an advantage in the environment since each replicon could have a different host range giving the plasmid a bigger chance of being horizontally transferred in the bacterial community.

Supporting information

S1 Fig. Computer-predicted secondary structure of the ctRNA. A. Minimum free energy and centroid secondary structure of ctRNA$_{repABC}$. B. Minimum free energy structure of ctRNA$_{repC}$. C. Centroid secondary structure of ctRNA$_{repC.}$
(PDF)

S2 Fig. Sequence alignment of ctRNA. The black boxes correspond to the -10 and -35 conserved promotor region; the sequence underlined corresponds to the isolated ctRNA from plasmids pRetCFN42d and pRmeGr4a; the bold G corresponds to +1 of pRetCFN42d ctRNA.
(PDF)

S3 Fig. Plasmid profiles of *S. meliloti* 2011 harboring pKrepC, pGrepABC or both by Eckhardt-like gels.
(TIFF)

S4 Fig. Plasmid profiles of *S. meliloti* LPU88, *S. meliloti* LPU88 (pKrepC) and *S. meliloti* LPU88 (pGrepABC) strains in Eckhardt-like gels. The figure shows the plasmid profile of strain LPU88 before and after receiving the constructed plasmids.
(TIFF)

S5 Fig. Plasmid profiles of *S. meliloti* 2011 harboring pKrepC2, pKrepC2-truncated by Eckhardt-like gels.
(TIFF)

S1 Table. Primers used in this work.
(XLSX)

S1 Raw images.
(PDF)

Acknowledgments

A.Lu. and L.G.C. are fellows of CONICET. M.F.D.P, A.La., G.T.T. and M.P are members of the Research Career of CONICET.

Author Contributions

Conceptualization: Abril Luchetti, Lucas G. Castellani, Gonzalo Torres Tejerizo, Mariano Pistorio.

Data curation: Abril Luchetti, Lucas G. Castellani, Andrés Martin Toscani, Gonzalo Torres Tejerizo, Mariano Pistorio.

Formal analysis: Abril Luchetti, Lucas G. Castellani, Andrés Martin Toscani, Antonio Lagares, María Florencia Del Papa, Gonzalo Torres Tejerizo, Mariano Pistorio.

Funding acquisition: Gonzalo Torres Tejerizo, Mariano Pistorio.

Investigation: Abril Luchetti, Lucas G. Castellani, Antonio Lagares, María Florencia Del Papa, Gonzalo Torres Tejerizo, Mariano Pistorio.

Methodology: Abril Luchetti, Lucas G. Castellani, Andrés Martin Toscani, Antonio Lagares, María Florencia Del Papa, Gonzalo Torres Tejerizo, Mariano Pistorio.

Project administration: Gonzalo Torres Tejerizo, Mariano Pistorio.

Resources: Gonzalo Torres Tejerizo, Mariano Pistorio.

Software: Gonzalo Torres Tejerizo, Mariano Pistorio.

Supervision: Gonzalo Torres Tejerizo, Mariano Pistorio.

Validation: Abril Luchetti, Gonzalo Torres Tejerizo, Mariano Pistorio.

Visualization: Gonzalo Torres Tejerizo, Mariano Pistorio.

Writing – original draft: Abril Luchetti, Lucas G. Castellani, Andrés Martin Toscani, Antonio Lagares, María Florencia Del Papa, Gonzalo Torres Tejerizo, Mariano Pistorio.

Writing – review & editing: Abril Luchetti, Lucas G. Castellani, Andrés Martin Toscani, Antonio Lagares, María Florencia Del Papa, Gonzalo Torres Tejerizo, Mariano Pistorio.

References

1. Smets BF, Barkay T. Horizontal gene transfer: perspectives at a crossroads of scientific disciplines. Nat Rev Microbiol. 2005; 3: 675–8. https://doi.org/10.1038/nrmicro1253 PMID: 16145755
2. Jurėnas D, Fraikin N, Goormaghtigh F, Van Melderen L. Biology and evolution of bacterial toxin–antitoxin systems. Nat Rev Microbiol. 2022; 20: 335–350. https://doi.org/10.1038/s41579-021-00661-1 PMID: 34975154

3. Salje J. Plasmid segregation: how to survive as an extra piece of DNA. Crit Rev Biochem Mol Biol. 2010; 45: 296–317. https://doi.org/10.3109/10409238.2010.494657 PMID: 20586577
4. Field CM, Summers DK. Multicopy plasmid stability: revisiting the dimer catastrophe. J Theor Biol. 2011; 291: 119–27. https://doi.org/10.1016/j.jtbi.2011.09.006 PMID: 21945338
5. Bañuelos-Vazquez LA, Torres Tejerizo G, Brom S. Regulation of conjugative transfer of plasmids and integrative conjugative elements. Plasmid. 2017; 91: 82–89. https://doi.org/10.1016/j.plasmid.2017.04.002 PMID: 28438469
6. García-de los Santos A, Brom S, Romero D. Rhizobium plasmids in bacteria-legume interactions. World J Microbiol Biotechnol. 1996; 12: 119–125. https://doi.org/10.1007/BF00364676 PMID: 24415159
7. Mercado-Blanco J. Plasmids in Rhizobia: The Role of Nonsymbiotic Plasmids. Mol Plant-Microbe Interact. 1996; 9: 535. https://doi.org/10.1094/MPMI-9-0535
8. Lagares A, Sanjuán J, Pistorio M. The Plasmid Mobilome of the Model Plant-Symbiont Sinorhizobium meliloti: Coming up with New Questions and Answers. Microbiol Spectr. 2014;2. https://doi.org/10.1128/microbiolspec.PLAS-0005-2013 PMID: 26104371
9. Torres Tejerizo G, Del Papa MF, Draghi W, Lozano M, Giusti M de los Á, Martini C, et al. First genomic analysis of the broad-host-range Rhizobium sp. LPU83 strain, a member of the low-genetic diversity Oregon-like Rhizobium sp. group. J Biotechnol. 2011; 155: 3–10. https://doi.org/10.1016/j.jbiotec.2011.01.011 PMID: 21329739
10. Hynes MF, Simon R, Müller P, Niehaus K, Labes M, Pühler A. The two megaplasmids of Rhizobium meliloti are involved in the effective nodulation of alfalfa. Mol Gen Genet MGG. 1986; 202: 356–362. https://doi.org/10.1007/BF00333262
11. Tabata S, Hooykaas PJ, Oka A. Sequence determination and characterization of the replicator region in the tumor-inducing plasmid pTiB6S3. J Bacteriol. 1989; 171: 1665–72. https://doi.org/10.1128/jb.171.3.1665-1672.1989 PMID: 2537824
12. Mazur A, Koper P. Rhizobial plasmids—replication, structure and biological role. Open Life Sci. 2012; 7: 571–586. https://doi.org/10.2478/s11535-012-0058-8
13. Cevallos MA, Cervantes-Rivera R, Gutiérrez-Ríos RM. The repABC plasmid family. Plasmid. 2008; 60: 19–37. https://doi.org/10.1016/j.plasmid.2008.03.001 PMID: 18433868
14. Cevallos MA, Porta H, Izquierdo J, Tun-Garrido C, García-de-los-Santos A, Dávila G, et al. Rhizobium etli CFN42 contains at least three plasmids of the repABC family: a structural and evolutionary analysis. Plasmid. 2002; 48: 104–116. https://doi.org/10.1016/s0147-619x(02)00119-1 PMID: 12383728
15. Ramírez-Romero MA, Soberón N, Pérez-Oseguera A, Téllez-Sosa J, Cevallos MA. Structural elements required for replication and incompatibility of the Rhizobium etli symbiotic plasmid. J Bacteriol. 2000; 182: 3117–3124. https://doi.org/10.1128/JB.182.11.3117-3124.2000 PMID: 10809690
16. Bartosik D, Baj J, Piechucka E, Waker E, Wlodarczyk M. Comparative characterization of repABC-type replicons of Paracoccus pantotrophus composite plasmids. Plasmid. 2002; 48: 130–41. https://doi.org/10.1016/s0147-619x(02)00100-2 PMID: 12383730
17. Mazur A, Majewska B, Stasiak G, Wielbo J, Skorupska A. repABC-based replication systems of Rhizobium leguminosarum bv. trifolii TA1 plasmids: Incompatibility and evolutionary analyses. Plasmid. 2011; 66: 53–66. https://doi.org/10.1016/j.plasmid.2011.04.002 PMID: 21620885
18. Bartosik D, Baj J, Wlodarczyk M. Molecular and functional analysis of pTAV320, a repABC-type replicon of the Paracoccus versutus composite plasmid pTAV1. Microbiology. 1998;144 (Pt 1: 3149–3157. https://doi.org/10.1099/00221287-144-11-3149 PMID: 9846751
19. MacLellan SR, Smallbone LA, Sibley CD, Finan TM. The expression of a novel antisense gene mediates incompatibility within the large repABC family of α-proteobacterial plasmids. Mol Microbiol. 2004; 55: 611–623. https://doi.org/10.1111/j.1365-2958.2004.04412.x PMID: 15659174
20. Venkova-Canova T, Soberón NE, Ramírez-Romero MA, Cevallos MA. Two discrete elements are required for the replication of a repABC plasmid: an antisense RNA and a stem-loop structure. Mol Microbiol. 2004; 54: 1431–1444. https://doi.org/10.1111/j.1365-2958.2004.04366.x PMID: 15554980
21. Cervantes-Rivera R, Romero-López C, Berzal-Herranz A, Cevallos MA. Analysis of the mechanism of action of the antisense RNA that controls the replication of the repABC plasmid p42d. J Bacteriol. 2010; 192: 3268–78. https://doi.org/10.1128/JB.00118-10 PMID: 20435728
22. Chai Y, Winans SC. A small antisense RNA downregulates expression of an essential replicase protein of an Agrobacterium tumefaciens Ti plasmid. Mol Microbiol. 2005; 56: 1574–85. https://doi.org/10.1111/j.1365-2958.2005.04636.x PMID: 15916607
23. Koper P, Żebracki K, Marczak M, Skorupska A, Mazur A. RepB proteins of the multipartite Rhizobium leguminosarum bv. trifolii genome discriminate between centromere-like parS sequences for plasmid

segregational stability. Mol Microbiol. 2016; 102: 446–466. https://doi.org/10.1111/mmi.13472 PMID: 27480612

24. Soberón N, Venkova-Canova T, Ramírez-Romero MA, Téllez-Sosa J, Cevallos MA. Incompatibility and the partitioning site of the repABC basic replicon of the symbiotic plasmid from Rhizobium etli. Plasmid. 2004; 51: 203–216. https://doi.org/10.1016/j.plasmid.2004.01.005 PMID: 15109827

25. MacLellan SR, Zaheer R, Sartor AL, MacLean AM, Finan TM. Identification of a megaplasmid centromere reveals genetic structural diversity within the repABC family of basic replicons. Mol Microbiol. 2006; 59: 1559–1575. https://doi.org/10.1111/j.1365-2958.2006.05040.x PMID: 16468995

26. Czarnecki J, Chapkauskaitse E, Bos J, Sentkowska D, Wawrzyniak P, Wyszyńska A, et al. Differential Localization and Functional Specialization of parS Centromere-Like Sites in repABC Replicons of Alphaproteobacteria. Appl Environ Microbiol. 2022; 88: e0020722. https://doi.org/10.1128/aem.00207-22 PMID: 35389251

27. Cervantes-Rivera R, Pedraza-López F, Pérez-Segura G, Cevallos MA. The replication origin of a repABC plasmid. BMC Microbiol. 2011; 11: 158. https://doi.org/10.1186/1471-2180-11-158 PMID: 21718544

28. Pinto UM, Flores-Mireles AL, Costa ED, Winans SC. RepC protein of the octopine-type Ti plasmid binds to the probable origin of replication within repC and functions only in cis. Mol Microbiol. 2011; 81: 1593–1606. https://doi.org/10.1111/j.1365-2958.2011.07789.x PMID: 21883520

29. Pérez-Oseguera Á, Cevallos MA. RepA and RepB exert plasmid incompatibility repressing the transcription of the repABC operon. Plasmid. 2013; 70: 362–376. https://doi.org/10.1016/j.plasmid.2013.08.001 PMID: 24016735

30. Rivera-Urbalejo A, Pérez-Oseguera Á, Carreón-Rodríguez OE, Cevallos MA. Mutations in an antisense RNA, involved in the replication control of a repABC plasmid, that disrupt plasmid incompatibility and mediate plasmid speciation. Plasmid. 2015; 78: 48–58. https://doi.org/10.1016/j.plasmid.2015.01.004 PMID: 25644116

31. Zebracki K, Koper P, Marczak M, Skorupska A, Mazur A. Plasmid-encoded RepA proteins specifically autorepress individual repABC operons in the multipartite Rhizobium leguminosarum bv. trifolii genome. PLoS One. 2015; 10: 1–24. https://doi.org/10.1371/journal.pone.0131907 PMID: 26147968

32. Mercado-Blanco J, Olivares J. The Large Nonsymbiotic Plasmid pRmeGR4a of Rhizobium meliloti GR4 Encodes a Protein Involved in Replication That Has Homology with the RepC Protein of Agrobacterium Plasmids. Plasmid. 1994; 32: 75–79. https://doi.org/10.1006/plas.1994.1046 PMID: 7991675

33. Izquierdo J, Venkova-Canova T, Ramírez-Romero MA, Téllez-Sosa J, Hernández-Lucas I, Sanjuan J, et al. An antisense RNA plays a central role in the replication control of a repC plasmid. Plasmid. 2005; 54: 259–77. https://doi.org/10.1016/j.plasmid.2005.05.003 PMID: 16005966

34. Barran LR, Ritchot N, Bromfield ES. Sinorhizobium meliloti plasmid pRm1132f replicates by a rolling-circle mechanism. J Bacteriol. 2001; 183: 2704–8. https://doi.org/10.1128/JB.183.8.2704-2708.2001 PMID: 11274136

35. Wheatley RM, Ramachandran VK, Geddes BA, Perry BJ, Yost CK, Poole PS. Role of O2 in the Growth of Rhizobium leguminosarum bv. viciae 3841 on Glucose and Succinate. J Bacteriol. 2017;199. https://doi.org/10.1128/JB.00572-16 PMID: 27795326

36. Pistorio M, Giusti MA, Del Papa MF, Draghi WO, Lozano MJ, Torres Tejerizo G, et al. Conjugal properties of the Sinorhizobium meliloti plasmid mobilome. FEMS Microbiol Ecol. 2008; 65: 372–382. https://doi.org/10.1111/j.1574-6941.2008.00509.x PMID: 18537840

37. Pistorio M, Torres Tejerizo GA, Del Papa MF, de los Angeles Giusti M, Lozano M, Lagares A. rptA, a novel gene from Ensifer (Sinorhizobium) meliloti involved in conjugal transfer. FEMS Microbiol Lett. 2013; 345: 22–30. https://doi.org/10.1111/1574-6968.12177 PMID: 23672494

38. Castellani LG, Nilsson JF, Wibberg D, Schlüter A, Pühler A, Brom S, et al. Insight into the structure, function and conjugative transfer of pLPU83a, an accessory plasmid of Rhizobium favelukesii LPU83. Plasmid. 2019; 103: 9–16. https://doi.org/10.1016/j.plasmid.2019.03.004 PMID: 30928704

39. Torres Tejerizo G, Florencia Del Papa M, de los Ángeles Giusti M, Draghi W, Lozano M, Lagares A, et al. Characterization of extrachromosomal replicons present in the extended host range Rhizobium sp. LPU83. Plasmid. 2010; 64: 177–185. https://doi.org/10.1016/j.plasmid.2010.07.004 PMID: 20621118

40. Torres Tejerizo G, Pistorio M, Althabegoiti MJ, Cervantes L, Wibberg D, Schlüter A, et al. Rhizobial plasmid pLPU83a is able to switch between different transfer machineries depending on its genomic background. FEMS Microbiol Ecol. 2014; 88: 565–578. https://doi.org/10.1111/1574-6941.12325 PMID: 24646299

41. Pistorio M, Del Papa MF, Balagué LJ, Lagares A. Identification of a transmissible plasmid from an Argentine Sinorhizobium meliloti strain which can be mobilised by conjugative helper functions of the

European strain S. meliloti GR4. FEMS Microbiol Lett. 2003; 225: 15–21. https://doi.org/10.1016/S0378-1097(03)00454-3 PMID: 12900015

42. de los ángeles Giusti M, Pistorio M, Lozano MJ, Torres Tejerizo GA, Salas ME, Martini MC, et al. Genetic and functional characterization of a yet-unclassified rhizobial Dtr (DNA-transfer-and-replication) region from a ubiquitous plasmid conjugal system present in Sinorhizobium meliloti, in Sinorhizobium medicae, and in other nonrhizobial Gram-negativ. Plasmid. 2012; 67: 199–210. https://doi.org/10.1016/j.plasmid.2011.12.010 PMID: 22233546

43. Miller JH. Experiments in molecular genetics. Cold Spring Harb Lab Cold Spring Harb NY. 1971.

44. Beringer JE. R factor transfer in Rhizobium leguminosarum. J Gen Microbiol. 1974; 84: 188–198. https://doi.org/10.1099/00221287-84-1-188 PMID: 4612098

45. Simon R, Priefer U, Pühler A. A Broad Host Range Mobilization System for In Vivo Genetic Engineering: Transposon Mutagenesis in Gram Negative Bacteria. Bio/Technology. 1983; 1: 784–791. https://doi.org/10.1038/nbt1183-784

46. Schäfer A, Tauch A, Jäger W, Kalinowski J, Thierbach G, Pühler A. Small mobilizable multi-purpose cloning vectors derived from the Escherichia coli plasmids pK18 and pK19: selection of defined deletions in the chromosome of Corynebacterium glutamicum. Gene. 1994; 145: 69–73. https://doi.org/10.1016/0378-1119(94)90324-7 PMID: 8045426

47. Kovach ME, Elzer PH, Hill DS, Robertson GT, Farris MA, Roop RM, et al. Four new derivatives of the broad-host-range cloning vector pBBR1MCS, carrying different antibiotic-resistance cassettes. Gene. 1995; 166: 175–6. https://doi.org/10.1016/0378-1119(95)00584-1 PMID: 8529885

48. Seemann T. Prokka: rapid prokaryotic genome annotation. Bioinformatics. 2014; 30: 2068–9. https://doi.org/10.1093/bioinformatics/btu153 PMID: 24642063

49. Aziz RK, Bartels D, Best AA, DeJongh M, Disz T, Edwards RA, et al. The RAST Server: rapid annotations using subsystems technology. BMC Genomics. 2008; 9: 75. https://doi.org/10.1186/1471-2164-9-75 PMID: 18261238

50. Sullivan MJ, Petty NK, Beatson SA. Easyfig: a genome comparison visualizer. Bioinformatics. 2011; 27: 1009–10. https://doi.org/10.1093/bioinformatics/btr039 PMID: 21278367

51. Eckhardt T. A rapid method for the identification of plasmid desoxyribonucleic acid in bacteria. Plasmid. 1978; 1: 584–588. https://doi.org/10.1016/0147-619x(78)90016-1 PMID: 107540

52. Wheatcroft R. Changes in the Rhizobium meliloti Genome and the Ability to Detect Supercoiled Plasmids During Bacteroid Development. Mol Plant-Microbe Interact. 1990; 3: 9. https://doi.org/10.1094/MPMI-3-009

53. Sambrook J, Fritsch EF, Maniatis T. Molecular Cloning: A Laboratory Manual (Second Edition). 2nd ed. Cold Spring Harbor, N.Y.: Cold Spring Harbor Laboratory Press; 1989.

54. Kado CI. Proceedings of the International Symposium on Plasmid Biology. Plasmid. 2007. p. 196. https://doi.org/10.1016/j.plasmid.2006.12.001

55. Pistorio M, Balagué LJ, Del Papa MF, Pich-Otero A, Lodeiro A, Hozbor DF, et al. Construction of a Sinorhizobium meliloti strain carrying a stable and non-transmissible chromosomal single copy of the green fluorescent protein GFP-P64L/S65T. FEMS Microbiol Lett. 2002; 214: 165–170. https://doi.org/10.1111/j.1574-6968.2002.tb11341.x PMID: 12351225

56. Watson RJ, Heys R. Replication regions of Sinorhizobium meliloti plasmids. Plasmid. 2006; 55: 87–98. https://doi.org/10.1016/j.plasmid.2005.08.003 PMID: 16202450

57. González V, Santamaría RI, Bustos P, Hernández-González I, Medrano-Soto A, Moreno-Hagelsieb G, et al. The partitioned Rhizobium etli genome: Genetic and metabolic redundancy in seven interacting replicons. Proc Natl Acad Sci. 2006; 103: 3834–3839. https://doi.org/10.1073/pnas.0508502103 PMID: 16505379

58. Summers DK, Beton CW, Withers HL. Multicopy plasmid instability: the dimer catastrophe hypothesis. Mol Microbiol. 1993; 8: 1031–8. https://doi.org/10.1111/j.1365-2958.1993.tb01648.x PMID: 8361350

59. Quintero V, Cevallos MA, Dávila G. A site-specific recombinase (RinQ) is required to exert incompatibility towards the symbiotic plasmid of Rhizobium etli. Mol Microbiol. 2002; 46: 1023–1032. https://doi.org/10.1046/j.1365-2958.2002.03205.x PMID: 12421308

60. Zhang J, Zhang Y, Zhu L, Suzuki M, Inouye M. Interference of mRNA function by sequence-specific endoribonuclease PemK. J Biol Chem. 2004; 279: 20678–20684. https://doi.org/10.1074/jbc.M314284200 PMID: 15024022

61. González V, Bustos P, Ramírez-Romero MA, Medrano-Soto A, Salgado H, Hernández-González I, et al. The mosaic structure of the symbiotic plasmid of Rhizobium etli CFN42 and its relation to other symbiotic genome compartments. Genome Biol. 2003;4. https://doi.org/10.1186/gb-2003-4-6-r36 PMID: 12801410

62. Young JPW, Crossman LC, Johnston AWB, Thomson NR, Ghazoui ZF, Hull KH, et al. The genome of Rhizobium leguminosarum has recognizable core and accessory components. Genome Biol. 2006; 7: R34. https://doi.org/10.1186/gb-2006-7-4-r34 PMID: 16640791

63. Martínez-Abarca F, Martínez-Rodríguez L, López-Contreras JA, Jiménez-Zurdo JI, Toro N. Complete genome sequence of the alfalfa symbiont Sinorhizobium/ Ensifer meliloti strain GR4. Genome Announc. 2013; 1: 4–5. https://doi.org/10.1128/genomeA.00174-12 PMID: 23409262

64. Kuhn S, Stiens M, Pühler A, Schlüter A. Prevalence of pSmeSM11a-like plasmids in indigenous Sinorhizobium meliloti strains isolated in the course of a field release experiment with genetically modified S. meliloti strains. FEMS Microbiol Ecol. 2008; 63: 118–31. https://doi.org/10.1111/j.1574-6941.2007.00399.x PMID: 18034835

65. Douarre PE, Mallet L, Radomski N, Felten A, Mistou MY. Analysis of COMPASS, a New Comprehensive Plasmid Database Revealed Prevalence of Multireplicon and Extensive Diversity of IncF Plasmids. Front Microbiol. 2020;11. https://doi.org/10.3389/fmicb.2020.00483 PMID: 32265894

66. Yamamoto S, Kiyokawa K, Tanaka K, Moriguchi K, Suzuki K. Novel toxin-antitoxin system composed of serine protease and AAA-ATPase homologues determines the high level of stability and incompatibility of the tumor-inducing plasmid pTiC58. J Bacteriol. 2009; 191: 4656–66. https://doi.org/10.1128/JB.00124-09 PMID: 19447904

67. Yamamoto S, Uraji M, Tanaka K, Moriguchi K, Suzuki K. Identification of pTi-SAKURA DNA region conferring enhancement of plasmid incompatibility and stability. Genes Genet Syst. 2007; 82: 197–206. https://doi.org/10.1266/ggs.82.197 PMID: 17660690

68. Stiens M, Schneiker S, Keller M, Kuhn S, Pühler A, Schlüter A. Sequence Analysis of the 144-Kilobase Accessory Plasmid pSmeSM11a, Isolated from a Dominant Sinorhizobium meliloti Strain Identified during a Long-Term Field Release Experiment. Appl Environ Microbiol. 2006; 72: 3662–3672. https://doi.org/10.1128/AEM.72.5.3662-3672.2006 PMID: 16672515
